# Supplementary material for: Understanding Artemisia cina Ethyl Acetate Extract’s Anthelmintic Effect on Haemonchus contortus Eggs and L3 Larvae: The Synergism of Peruvin Binary Mixtures
Source: Pathogens. 2024 Jun 16;13(6):509. doi: 10.3390/pathogens13060509 (PMC11206963; doi:10.3390/pathogens13060509)
Supplement: Supplementary file 1 [file pathogens-13-00509-s001.zip › pathogens-3027975-supplementary.pdf]

Understanding *Artemisia cina* Ethyl Acetate Extract's Anthelmintic Effect on *Haemonchus contortus* Eggs and L_3 Larvae: The Synergism of Peruvin Binary Mixtures

Luis David Arango-De-la Pava [1], Manasés González-Cortazar [2], Alejandro Zamilpa [2], Jorge Alfredo Cuéllar-Ordaz [1], Héctor Alejandro de la Cruz-Cruz [1], Rosa Isabel Higuera-Piedrahita [1,2,*] and Raquel López-Arellano [1,*]

1 Facultad de Estudios Superiores Cuautitlán, Universidad Nacional Autónoma de México, Cuautitlán 54714, Estado de México, Mexico; luis.david@cuautitlan.unam.mx (L.D.A.-D.-l.P.); jcuellar@unam.mx (J.A.C.-O.); delacruz@unam.mx (H.A.d.l.C.-C.)

2 Centro de Investigación Biomédica del Sur, Instituto Mexicano del Seguro Social, Xochitepec 62790, Morelos, Mexico; gmanases@hotmail.com (M.G.-C.); azamilpa_2000@yahoo.com.mx (A.Z.)

* Correspondence: rhiguera05@comunidad.unam.mx (R.I.H.-P.); lopezar@unam.mx (R.L.-A.); Tel.: +52-1-5554524827 (R.L.-A.)

Supplementary Material

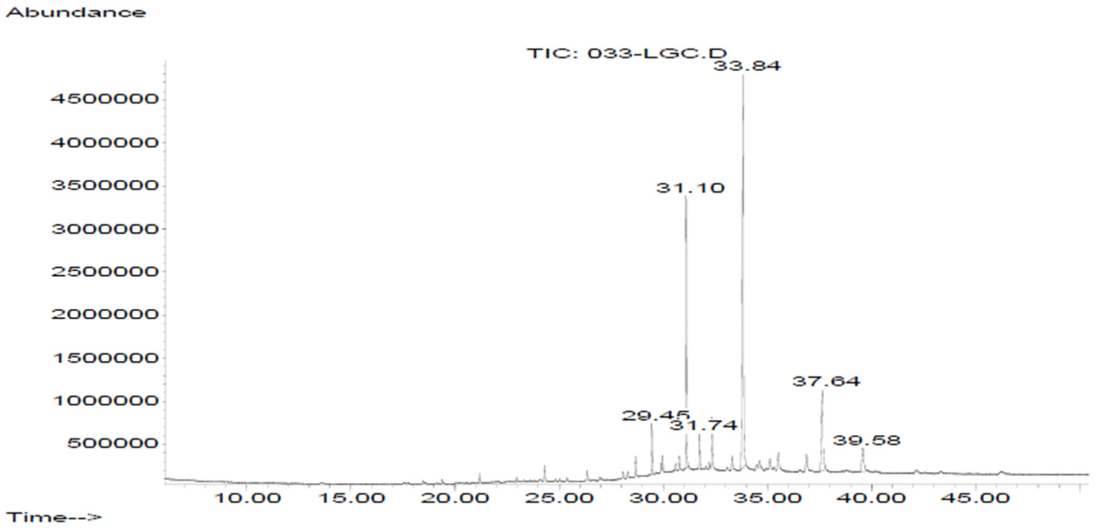

peak #	R.T. min	first scan	max scan	last scan	PK TY	peak height	corr. area	corr. % max.	% of total
1	29.450	3549	3558	3569	M	608451	15283564	7.50%	3.946%
2	31.105	3796	3810	3826	BB 2	3171628	82670471	40.57%	31.343%
3	31.736	3899	3906	3916	M	416056	11771847	5.78%	3.039%
4	33.838	4204	4226	4248	BB 2	4723968	203784668	100.00%	52.612%
5	37.641	4786	4805	4822	M4	950753	54055484	26.53%	3.956%
6	39.572	5083	5099	5122	M4	281208	19771821	9.70%	5.104%

Figure S1. GC-MS chromatogram of C1F3 isolated from *Artemisia cina* ethyl acetate extract.

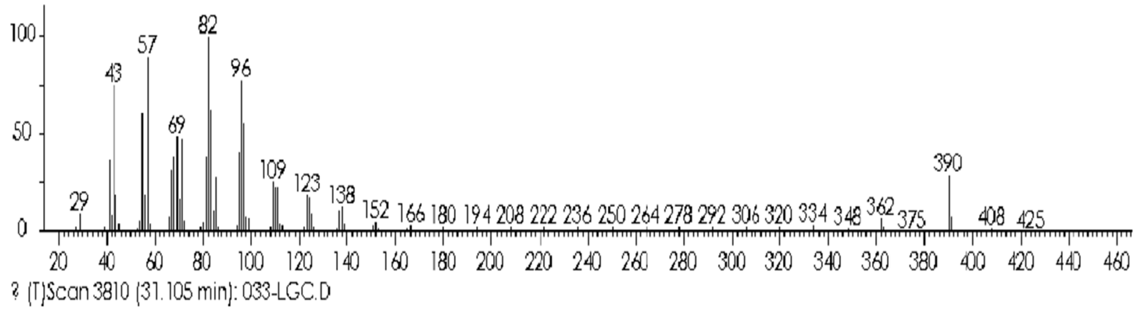

Figure S2. Mass spectra of 1-nonacosanol identified from *Artemisia cina* **ethyl acetate extract.**

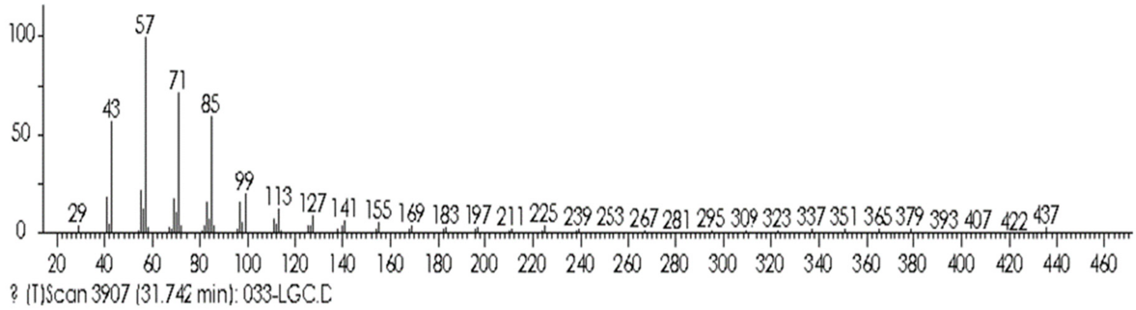

Figure S3. Mass spectra of hentriacontane identified from *Artemisia cina* ethyl acetate extract.

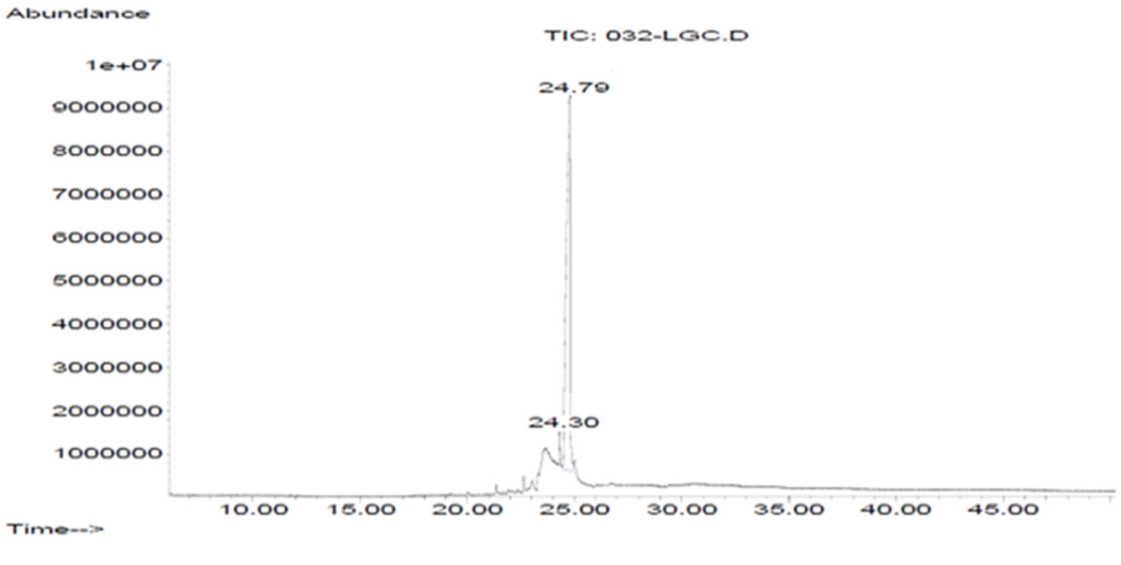

Signal : TIC

peak #	R.T. min	first scan	max scan	last scan	PK TY	peak height	corr. area	corr. % max.	% of total
1	24.300	2767	2774	2784	M	798164	22619630	6.35%	2.039%
2	24.786	2797	2848	2870	M7	8705518	1109214225	100.00%	97.961%

Figure S4. CG-MS chromatogram of cinic acid isolated from *Artemisia cina* ethyl acetate extract.

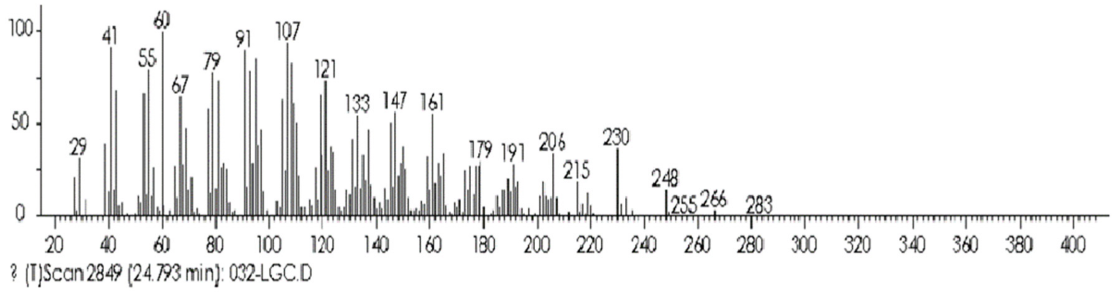

Figure S5. Cinic acid mass spectra isolated from *Artemisia cina* ethyl acetate extract.

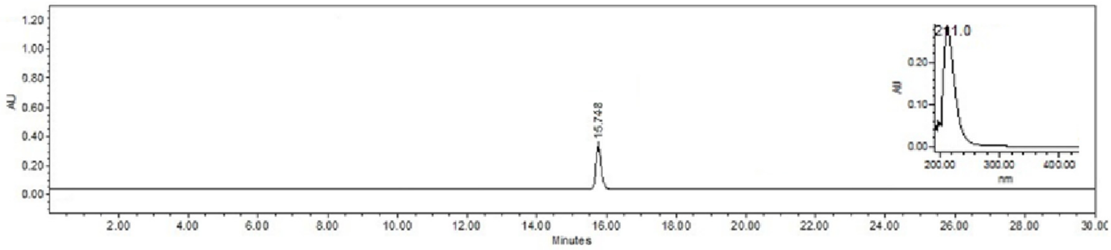

Figure S6. Chromatogram and UV-VIS spectra obtained by HPLC-DAD and viewed at 215 nm of cinic acid isolated from *Artemisia cina* ethyl acetate extract.

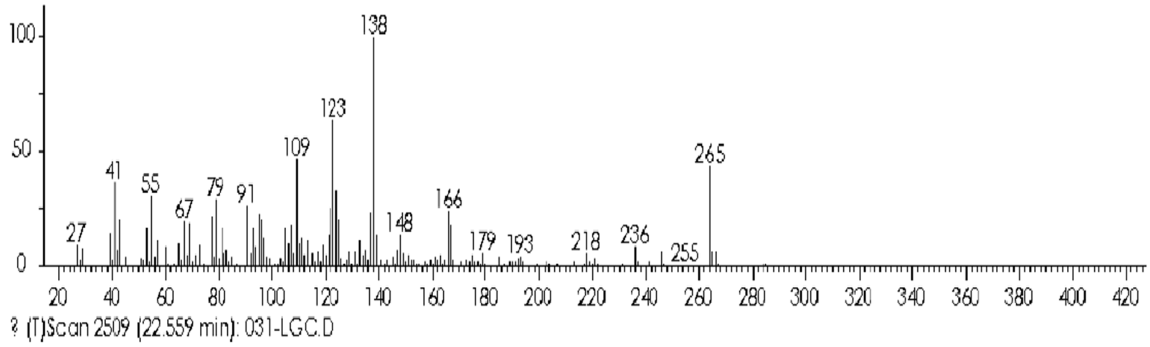

Figure S7. Peruvin mass spectra isolated from *Artemisia cina* ethyl acetate extract.

peak #	R.T. min	first scan	max scan	last scan	PK TY	peak height	corr. area	corr. % max.	% of total
1	22.526	2279	2504	2735	M	708814	656083589	100.00%	91.711%
2	24.306	2754	2775	2790	BB	2223391	59300522	9.04%	8.289%

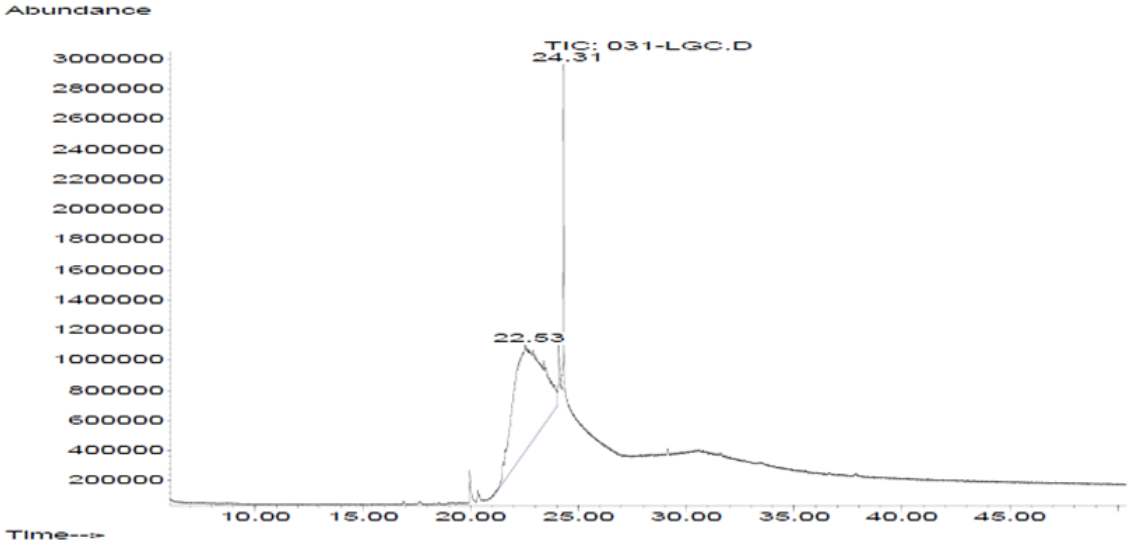

Figure S8. Peruvin CG-MS chromatogram isolated from *Artemisia cina* ethyl acetate extract.

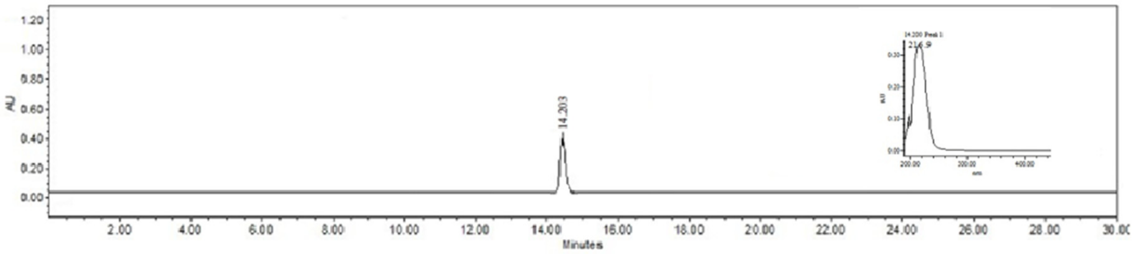

Figure S9. Chromatogram and UV-VIS spectra obtained by HPLC-DAD and viewed at 215 nm of peruvin isolated from *Artemisia cina* ethyl acetate extract.

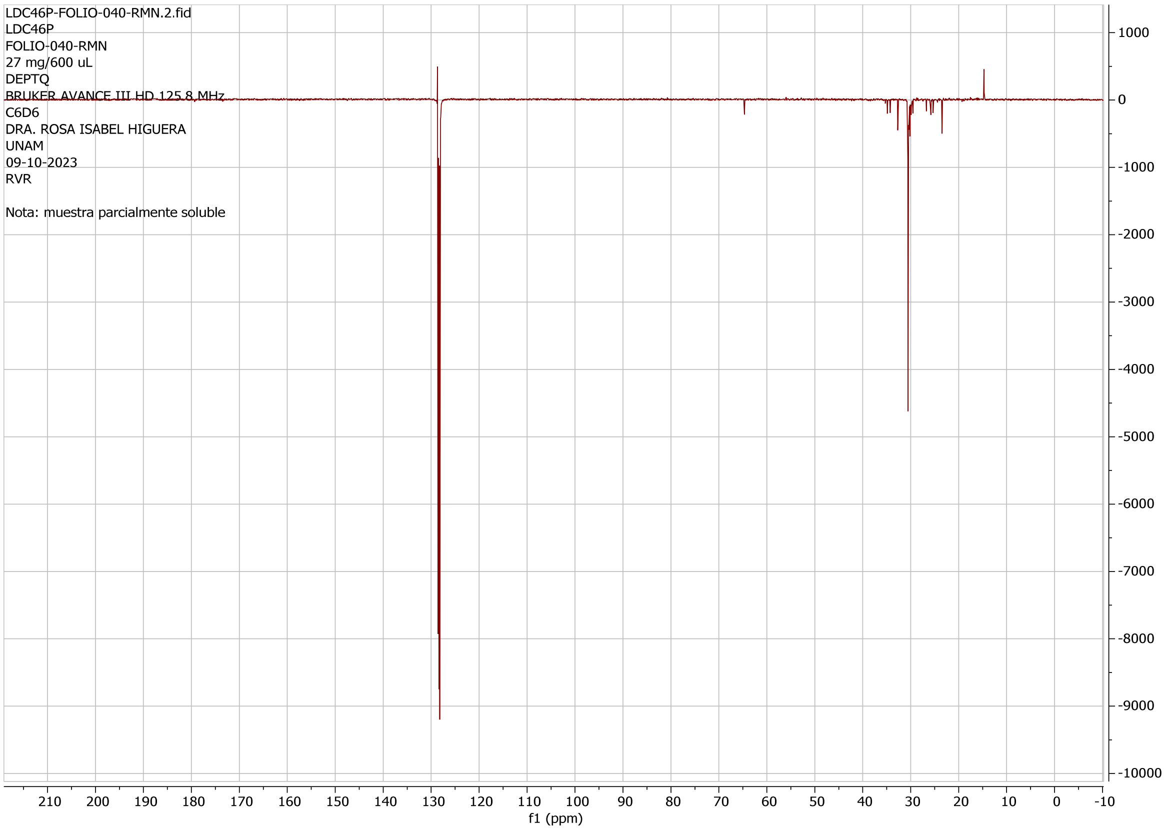

Figure S10. DEPTq spectra obtained at 500 MHz of C1F3 isolated from *Artemisia cina* ethyl acetate extract and dissolved in D_6H_6.

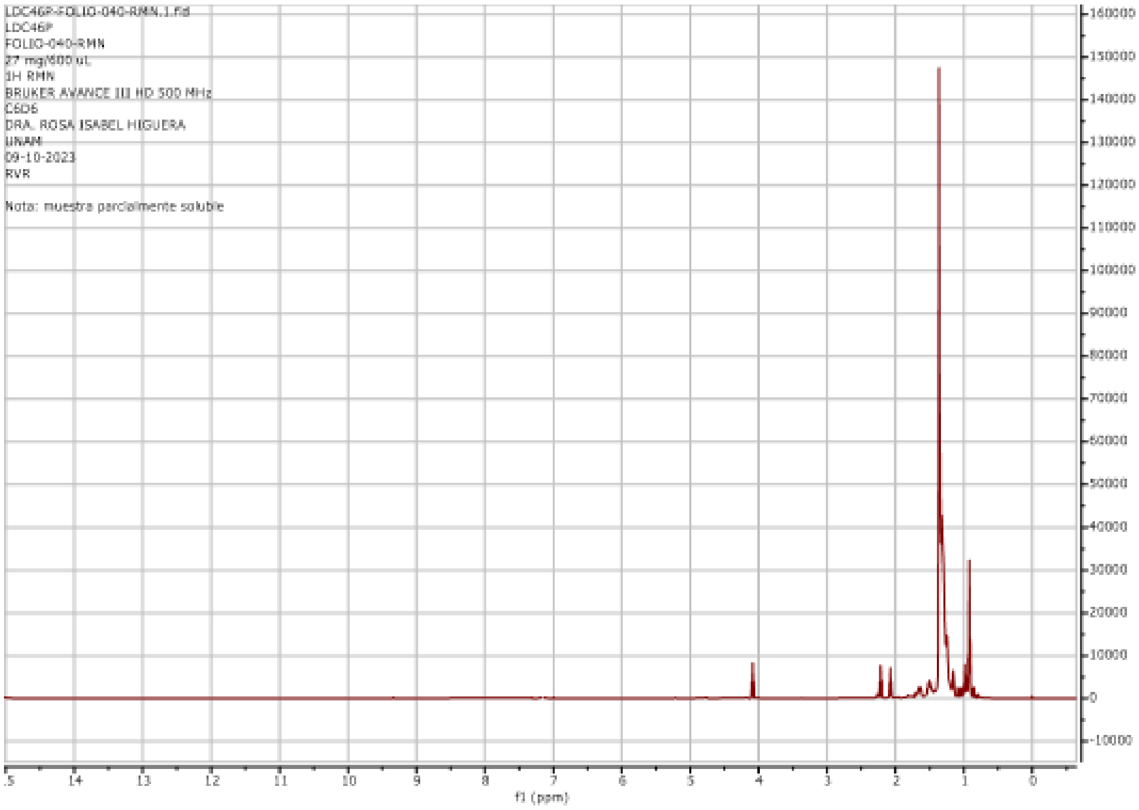

Figure S11. **^{1}H NMR spectra obtained at 500 MHz of C1F3 isolated from *Artemisia cina* ethyl acetate extract and dissolved in D_6H_6.**

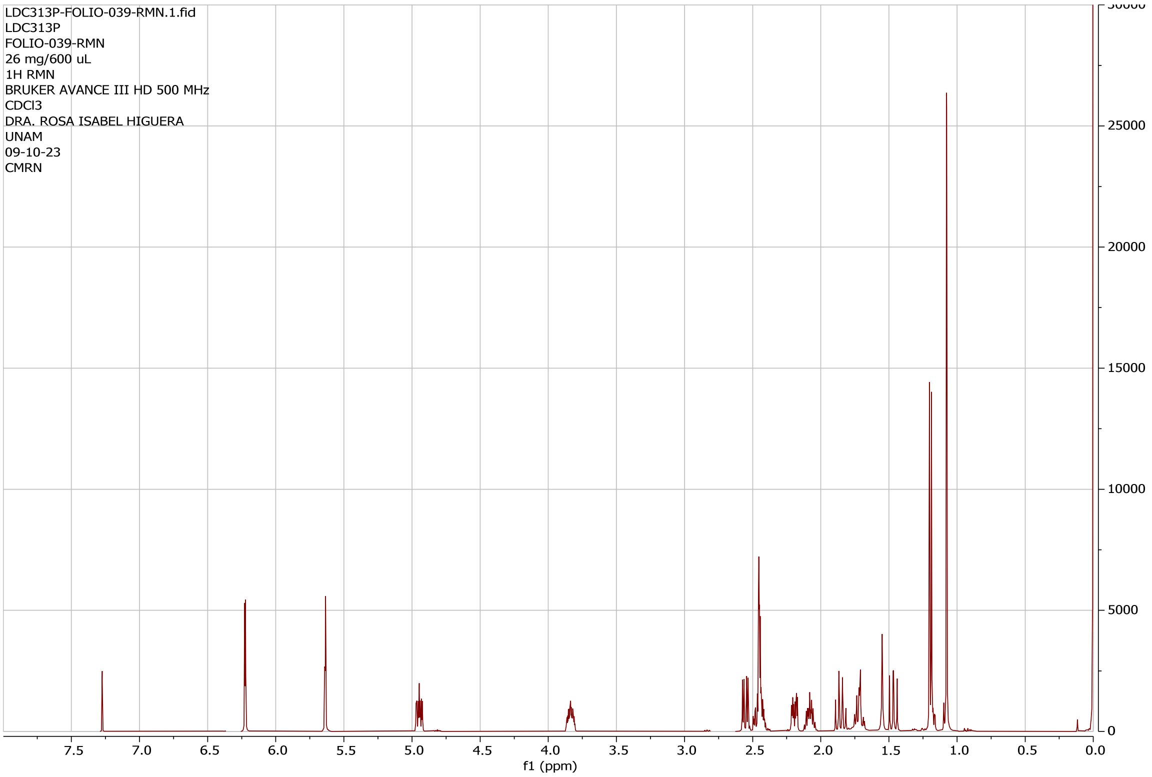

Figure S12. [1]**H NMR spectra obtained at 500 MHz of peruvin isolated from** ***Artemisia cina*** **ethyl acetate extract and dissolved in** CD_3Cl_3.

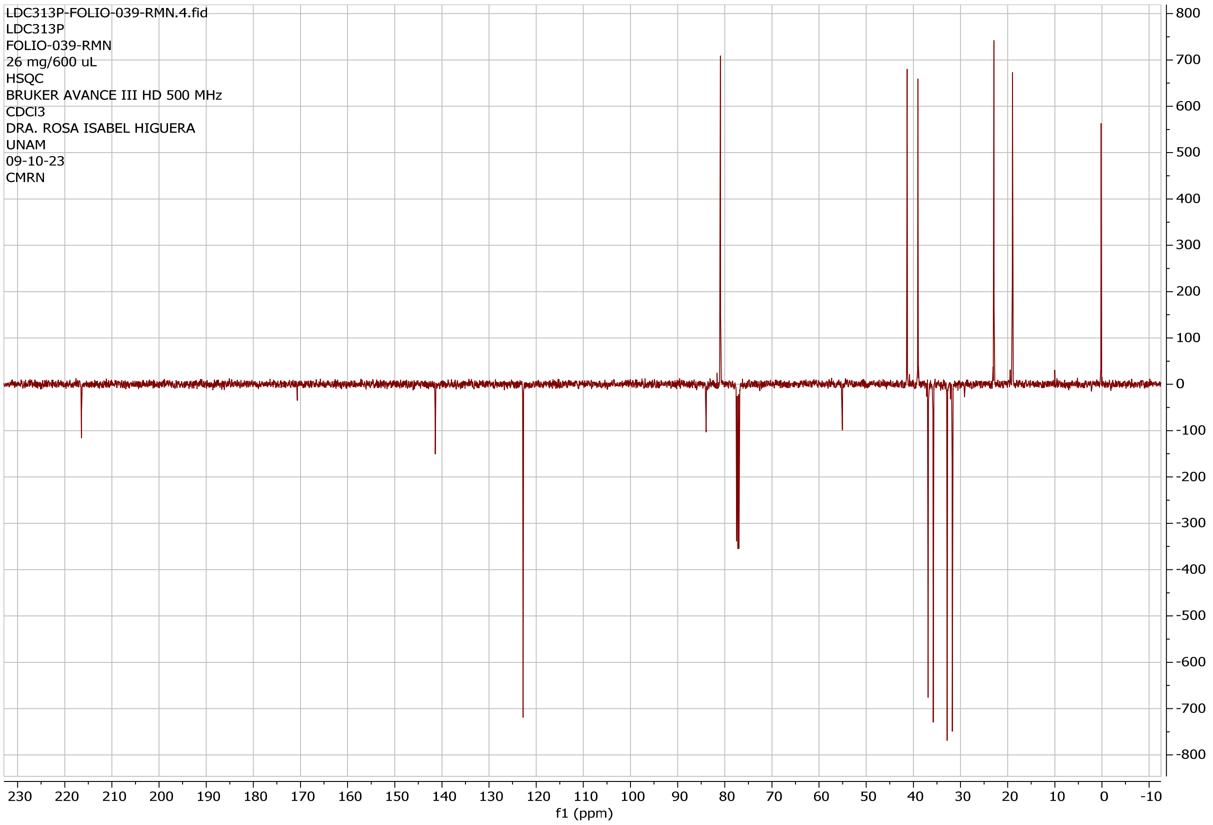

Figure S13. DEPTq spectra obtained at 500 MHz of peruvin isolated from *Artemisia cina* ethyl acetate extract and dissolved in CD_3COCD_3.

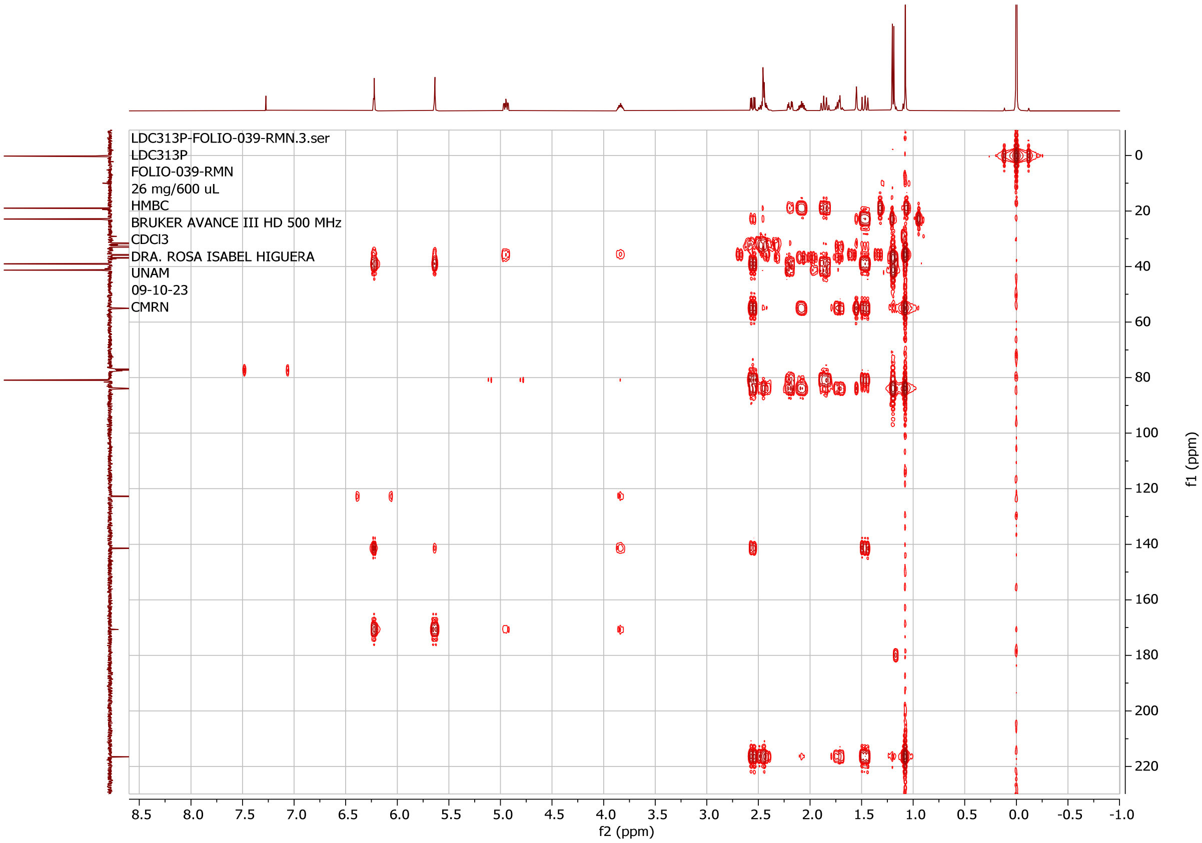

Figure S14. HMBC analysis of peruvin dissolved in CD_3Cl_3 and obtained at 500 MHz.
